# Eating within planetary boundaries - a cross-country analysis of iodine provision from the EAT-Lancet diet

**DOI:** 10.1038/s41538-025-00612-7

**Published:** 2025-11-24

**Authors:** Katie Nicol, Anne P. Nugent, Jayne V. Woodside, Kathryn H. Hart, Katie Lynch, Nicole Mangan, Sarah C. Bath

**Affiliations:** 1 Discipline of Nutrition, Exercise, Chronobiology and Sleep, Faculty of Health and Medical Sciences, University of Surrey, Guildford, England UK; 2 Institute for Global Food Security, School of Biological Sciences, Queen’s University Belfast, Belfast, Ireland UK; 3 Institute of Food and Health, School of Agriculture and Food Sciences, University College Dublin, Dublin, Ireland UK; 4 Centre for Public Health, School of Medicine, Dentistry and Biomedical Sciences, Queen’s University Belfast, Belfast, Ireland UK

**Keywords:** Nutrition, Thyroid gland

## Abstract

The EAT-Lancet Commission’s 2019 reference diet promotes health and environmental sustainability through predominantly plant-based foods, raising concerns about micronutrient adequacy, particularly iodine. This study evaluated the iodine content of the EAT-Lancet diet across sixteen countries using national food composition data. Iodine intake was modelled under three scenarios: (1) strict adherence to specified food items; (2) inclusion of a broader range of foods within each group; and (3) a vegan adaptation. In Scenario 1, dairy products, fish, and eggs were primary iodine sources, with intakes ranging from 42 µg/day (New Zealand) to 129 µg/day (United Kingdom), covering 28–85% of the adult requirements. Scenarios 2 and 3 showed higher iodine levels in countries using fortified bread, but most remained below adult and pregnancy requirements. These findings underscore the need to carefully evaluate iodine provision of plant-based dietary recommendations, particularly in countries without a fortification policy, to prevent iodine insufficiency.

## Introduction

In 2019, the EAT-Lancet Commission on Food, Planet, and Health published its framework for a planetary health reference diet^1^. The EAT-Lancet diet aims to reduce the incidence of non-communicable diseases and mortality globally while improving food system sustainability to help achieve the United Nation’s Sustainable Development Goals and the Paris Agreement^2^. It was a pioneering attempt to balance human nutritional needs with planetary sustainability, generating significant interest among academics, health professionals, policymakers, and the public^3^. The report gained widespread attention, shaping debates on sustainable diets, influencing policy decisions and becoming one of the most discussed scientific publications^4^.

The EAT-Lancet diet is predominantly plant-based, consisting of whole grains, fruits, vegetables, nuts, legumes, unsaturated oils, low to moderate amounts of seafood, poultry and dairy products, and no or low red meat or processed meat^1^. Despite this relatively low intake of animal-derived products, the EAT-Lancet Commission asserted that the diet would meet all essential nutrient requirements for all individuals older than two years. However, it is not clear whether the EAT-Lancet diet can provide adequate iodine to meet population needs, given the fact that plant-based foods have a low iodine concentration unless they are fortified^5^.

Iodine is an essential micronutrient that is required for thyroid hormone production, and therefore is critical for growth, brain development, and metabolic regulation^6^. Inadequate iodine intake can lead to a range of health issues, including goitre, hypothyroidism, and impaired cognitive development^7^, particularly if iodine deficiency is present during pregnancy and early life^8^. Given that iodine deficiency is the leading cause of preventable intellectual disability worldwide^9^, ensuring sufficient iodine intake is a major public health priority. To prevent iodine deficiency, many countries have implemented fortification strategies^10^, such as mandatory iodisation of salt or adding iodised salt to staple foods, including bread. However, policies differ significantly, and in countries like the UK and Ireland, where the use of iodised salt is not mandatory, population coverage remains low.

The EAT-Lancet Commission did not mention iodine in the report, nor did it include the use of iodised salt as a strategy to ensure adequate iodine intake^1^. Iodine-rich foods include fish, dairy products and eggs, which are limited in the EAT-Lancet diet. However, iodine content of these foods varies widely depending on geography and production methods. In particular, dairy products are not inherently rich in iodine; their iodine content is largely influenced by farming practices, such as the use of iodine-fortified cattle feed and disinfectants, which vary between countries^11^ and between conventional and organic systems^11^. One key issue is that the EAT-Lancet diet features low quantities of dairy products, the primary dietary source of iodine in many countries^10^. Given the importance of dairy products in iodine nutrition, reducing dairy product intake raises concerns about iodine adequacy^12^. This is especially true in countries that do not have iodine fortification polices, such as iodised salt provision or iodised salt added to bread products and therefore rely more on dietary sources (largely animal products) as sources of iodine.

Multiple studies have shown that reducing animal-sourced food intake can negatively affect micronutrient intake and status. Beal and colleagues^13,14^ demonstrated that an average of 35% of calories from animal-sourced foods is needed to ensure adequate micronutrient intake across populations^14,15^, far exceeding the 14% provided by animal-sourced foods in the EAT-Lancet diet^1^. While Beal et al. did not assess iodine specifically, their findings highlight the general risks of micronutrient inadequacy when reducing animal-sourced food intake.

Previous research has also shown that plant-based or environmentally conscious diets, typically characterised by reduced consumption of animal-sourced foods, tend to result in lower intakes of several key micronutrients, including iodine^5,16,17^. For example, a systematic review of dietary patterns aimed at reducing environmental impact found that such shifts consistently led to lower intakes of iodine, calcium, zinc, and vitamins A, B12, and D^17^.

Despite the growing recognition of the EAT-Lancet diet as a framework for promoting healthy diets within planetary boundaries, no studies have specifically examined its impact on iodine intake. To address this gap, the present study calculates the iodine content of the EAT-Lancet diet across different countries using country-specific food composition data. Three calculation scenarios were applied: Scenario 1, where food codes were closely matched to the foods listed in the EAT-Lancet diet; Scenario 2, where a wider range of food codes were included but within the overall proportions of each food group in the EAT-Lancet diet; and Scenario 3, which evaluates a vegan version of the diet. This analysis provides critical insight into whether the diet, in its current form, meets iodine requirements and informs potential modifications to ensure nutritional adequacy with regard to iodine.

## Results

A total of 37 publicly-available national food composition tables were identified^11,18,19^, but only the 16 that contained iodine-concentration data and an English translation were included in this analysis (Table 1). Of the countries with iodine-concentration data, the majority were European countries (*n* = 12), representation from Asia and Oceania is more limited, while no countries from Africa, North America, or South America had iodine-concentration data.

**Table 1:** National food composition tables with iodine-concentration data and an English translation used in this analysis

Country	Institution	Year published	Database
Australia	Food Standards Australia & New Zealand	2022	Australian Food Composition Database^52^
Denmark	National Food Institute, Technical University of Denmark	2022	Frida^53^
Estonia	National Institute for Health Development	2020	Nutridata^54^
Finland	Finnish Institute for Health and Welfare	2019	Fineli
France	Agency for Food, Environmental, Occupational Health & Safety	2020	CIQUAL French food composition table^55^
Iceland	Matís	2009	The Icelandic Food Composition Database (ISGEM)^56^
Italy	Banca Dati di Composizione degli Alimenti per Studi Epidemiologici	2022	Food Composition Database for Epidemiological Studies in Italy^57^
Japan	Ministry of Education, Culture, Sports, Science, and Technology, Japan	2015	Standard Tables of Food Composition in Japan^58^
Netherlands	Institute of Public Health and the Environment	2021	Nederlands Voedingsstoffenbestand NEVO)^59^
New Zealand	The New Zealand Institute for Plant and Food Research & the Ministry of Health	2022	New Zealand FOODfiles^60^
Norway	Norwegian Food Safety Authority, University of Oslo	2022	Norwegian Food Composition Tables^61^
Slovakia	Ministry of Agriculture and Rural Development of the Slovak Republic	2013	Slovak Food Composition Database^62^
Spain	Consortium BEDCA & Agencia Española de Seguridad Alimentaria	2007	Spanish Food Composition Database (BEDCA)^63^
South Korea	Rural Development Administration	2019	Korean Food Composition Table^64^
Sweden	Swedish National Food Agency	2022	Swedish Food Composition Database^65^
UK	Public Health England	2021	Composition of foods integrated dataset^27^

Scenario 1: Food codes matched to the EAT-Lancet diet. Scenario 2: Adapted EAT-Lancet diet to include a wider variety of food codes while maintaining the overall proportions from each group. Scenario 3: Vegan version of the EAT-Lancet diet.

### Scenario 1: iodine provision from the original EAT-Lancet diet

**Fig. 1:**
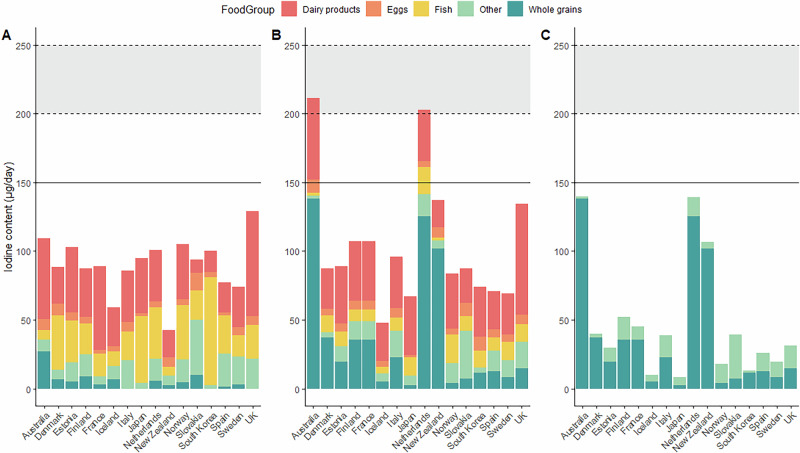
Total daily iodine provided by the EAT-Lancet diet. **A** Scenario 1 **B** Scenario 2 **C** Scenario 3. The horizontal line indicates adult RNI (150 µg/day), and the horizontal dashed lines and shaded grey area indicates the range of RNIs for pregnancy (200–250 µg/day). Scenario 1: Food codes matched to the EAT-Lancet diet. Scenario 2: Adapted EAT-Lancet diet to include a wider variety of food codes while maintaining the overall proportions from each group. Scenario 3: Vegan version of the EAT-Lancet diet.

The estimated iodine content of the EAT-Lancet diet (Scenario 1) ranged from 42 µg/day in New Zealand to 129 µg/day in the UK (Table 2; Fig. 1), which is 28-85% of the recommended intake for adults (at 150 µg/day^20–22^, Table 3). When considering the range of intake suggested for each food category, the iodine provision in Scenario 1 could be as low as 3 µg/day (2% of adult RNI) or as high as 315 µg/day (210% of daily adult RNI; Supplementary Table 3). Regardless of country, the calculated provision of iodine in the EAT-Lancet diet was below the Reference Nutrient Intake (RNI) for pregnancy, the diet would provide just 17–64% of the pregnancy recommended intake of iodine (220–250 µg/day; Table 3)^20,21^ and therefore would place individuals at risk of iodine deficiency during pregnancy. The main contributors to the overall iodine content of the EAT-Lancet diet were dairy products (10.0–76.3 µg iodine/day), fish (6.2–78.8 µg/day) and eggs (2.2–12.6 µg/day; Table 2). Whole grains contributed 27.1 µg iodine/day in Australia, and vegetables contributed 12.5 µg/day in Spain (Table 2).

**Table 2:** Calculations of the iodine content of each food group in the EAT-Lancet diet (µg/d) by country and Scenario

		Whole grains	Tubers	Vegetables	Fruits	Dairy foods	Beef & lamb	Pork	Chicken & other poultry	Eggs	Fish	Legumes	Added fats	Added sugar	Total
EAT – Lancet diet (g/d)		232	50	300	200	250	7	7	29	13	28	125	51.8	31	NA
Country	Scenario	Iodine from food (µg/d)													
Australia	Sc. 1	27.1	0.0	3.6	0.3	59.0	0.0	0.1	0.0	7.5	7.0	1.0	0.1	3.6	109
	Sc. 2	138.2	0.1	1.2	0.1	59.3	0.1	0.1	0.5	10.0	1.9	0.1	0.0	0.0	212
Denmark	Sc. 1	6.9	0.6	2.0	0.2	26.8	0.0	0.1	0.3	8.5	39.4	2.6	0.2	0.9	89
	Sc. 2	37.4	0.2	1.6	0.2	29.8	0.1	0.1	1.2	4.4	12.4	0.2	0.0	0.0	88
Estonia	Sc. 1	5.2	0.5	2.3	3.3	47.5	0.2	0.1	1.7	5.6	30.7	4.8	0.2	0.7	103
	Sc. 2	19.7	0.6	5.1	3.7	42.2	0.2	0.2	0.9	5.7	10.4	0.6	0.0	0.0	89
Finland	Sc. 1	9.1	0.5	3.0	1.4	35.5	0.2	0.2	4.4	4.8	22.0	4.9	0.2	1.2	87
	Sc. 2	35.5	1.4	5.2	2.2	43.3	0.3	0.2	3.6	6.3	8.4	0.7	0.0	0.1	107
France	Sc. 1	3.1	0.6	3.8	0.0	60.8	0.1	0.1	0.1	2.7	16.6	0.5	0.2	0.4	89
	Sc. 2	35.5	1.4	5.2	2.2	43.3	0.3	0.2	3.6	6.3	8.4	0.7	0.0	0.1	107
Iceland	Sc. 1	6.7	0.6	3.7	0.6	28.0	0.1	0.2	0.7	4.1	10.5	0.6	2.3	0.9	59
	Sc. 2	5.2	0.6	2.8	0.6	28.0	0.1	0.2	0.7	4.1	4.9	0.4	0.4	0.0	48
Italy	Sc. 1	0.0	0.5	3.0	6.7	37.5	0.7	0.4	1.7	6.9	20.6	7.4	0.3	0.0	86
	Sc. 2	22.9	0.5	3.0	10.0	37.5	0.8	0.4	2.0	6.9	9.9	2.2	0.0	0.0	96
Japan	Sc. 1	0.0	0.0	3.4	0.0	40.0	0.1	0.0	0.0	2.2	49.0	0.3	0.0	0.0	95
	Sc. 2	2.3	0.0	3.7	0.0	42.5	0.1	0.1	0.7	2.1	13.2	0.1	0.0	2.3	67
Netherlands	Sc. 1	5.6	1.3	5.7	5.0	37.3	0.2	0.1	1.7	4.1	37.4	1.5	0.1	0.7	101
	Sc. 2	125.5	1.3	7.2	5.0	37.3	0.3	0.2	1.6	4.3	19.8	0.5	0.0	0.0	203
New Zealand	Sc. 1	2.6	1.9	3.7	0.1	19.3	0.1	0.1	0.2	7.2	6.2	0.5	0.1	0.3	42
	Sc. 2	101.6	0.4	4.0	0.3	19.8	0.1	0.1	0.7	7.2	2.4	0.3	0.0	0.0	137
Norway	Sc. 1	4.6	0.5	5.1	4.0	40.0	0.1	0.0	0.3	4.4	39.6	5.8	0.0	0.6	105
	Sc. 2	4.0	0.8	7.4	4.5	40.1	0.2	0.0	0.5	4.7	20.5	1.2	0.0	0.0	84
Slovakia	Sc. 1	10.0	4.5	11.8	10.3	10.0	0.6	0.0	0.3	12.6	21.6	10.9	0.2	1.3	94
	Sc. 2	7.0	3.7	15.7	10.3	25.0	0.8	0.1	1.6	9.8	10.6	2.3	0.5	0.0	87
South Korea	Sc. 1	0.0	0.0	0.5	0.0	15.2	0.1	0.0	0.0	3.9	78.9	1.6	0.0	0.0	100
	Sc. 2	11.4	0.0	1.3	0.0	36.3	0.0	0.1	1.7	10.1	12.3	0.7	0.0	0.0	74
Spain	Sc. 1	1.3	1.3	12.5	2.7	21.5	0.1	0.2	0.5	2.6	27.8	6.2	0.2	0.2	77
	Sc. 2	12.4	1.1	8.2	2.7	28.0	0.3	0.1	1.5	5.7	9.8	1.3	0.0	0.0	71
Sweden	Sc. 1	3.0	0.7	3.6	2.7	29.5	2.6	0.1	0.1	5.5	15.7	10.0	0.1	0.4	74
	Sc. 2	8.3	1.4	6.4	1.1	29.8	0.3	0.1	0.3	5.6	13.4	2.5	0.0	0.0	69
UK	Sc. 1	0.0	0.0	6.4	4.7	76.3	0.6	0.0	1.5	6.5	24.5	6.6	0.6	1.4	129
	Sc. 2	15.0	0.6	7.7	6.0	80.4	0.8	0.5	1.7	7.2	12.5	1.8	0.0	0.0	134
Average	Sc. 1	5.3	0.8	6.4	2.6	36.5	0.4	0.1	0.8	5.6	28.0	3.7	0.3	0.6	91
	Sc. 2	36.9	0.8	5.8	3.0	38.4	0.3	0.2	1.6	6.2	10.8	1.0	0.1	0.3	105

Scenario 1: Food codes matched to the EAT-Lancet diet.

Scenario 2: Adapted EAT-Lancet diet to include a wider variety of food codes while maintaining the overall proportions from each group.

**Table 3:** Total iodine content of the EAT-Lancet diet by Scenario in each country, and the contribution to the RNI for adults and pregnant women

	Scenario 1			Scenario 2			Scenario 3		
	Iodine content (µg/day)	Contribution to adult RNI (%)^a^	Contribution to pregnancy RNI (%)^b^	Iodine content (µg/day)	Contribution to adult RNI (%)^a^	Contribution to pregnancy RNI (%)^b^	Iodine content (µg/day) Total	Contribution to adult RNI (%)^a^	Contribution to pregnancy RNI (%)^b^
Australia	109	73	44–55	212	141	85–106	140	93	56–70
Denmark	89	59	35–44	88	59	35–44	40	26	16–20
Estonia	103	69	41–52	89	60	36–45	30	20	12–15
Finland	87	58	35–44	120	80	48–60	45	30	18–23
France	89	59	36–45	107	71	43–54	45	30	18–23
Iceland	59	39	24–30	48	32	19–24	10	7	4–5
Italy	86	57	34–43	96	64	38–48	39	26	15–19
Japan	95	63	38–48	67	45	27–34	8	6	3–4
Netherlands	101	67	40–50	203	135	81–102	140	93	56–70
New Zealand	42	28	17–21	137	91	55–69	107	71	43–53
Norway	105	70	42–53	84	56	34–42	18	12	7–9
Slovakia	94	63	38–47	87	58	35-44	40	26	16–20
South Korea	100	67	40–50	75	50	30–38	13	9	5–7
Spain	77	51	31–39	70	47	28–35	26	17	10–13
Sweden	74	49	29–37	69	46	28–35	20	13	8–10
UK	129	85	51–64	134	89	54–67	31	21	12–16
Average	91	61	37–46	105	70	42–53	48	32	19–24

Scenario 1: Food codes matched to the EAT-Lancet diet.

Scenario 2: Adapted EAT-Lancet diet to include a wider variety of food codes while maintaining the overall proportions from each group.

Scenario 3: Vegan version of the EAT-Lancet diet.

^a^Iodine intake recommendations for adults: 150 µg/day^20–22^.

^b^Iodine intake recommendations for pregnancy: 200–250 µg/day^20–22^.

### Scenario 2: iodine provision from the adapted EAT-Lancet diet

After adapting the EAT-Lancet diet to include more varied foods within each food group in the calculations (Scenario 2), the estimated iodine content of the EAT-Lancet diet ranged from 48 µg/day in Iceland to 212 µg/day in Australia (Table 2; Fig. 1), which is 32–141% of the recommended intake for adults (at 150 µg/day^20–22^; Table 3). Depending on the intake range recommended for each food category, iodine provision in Scenario 2 could vary from as low as 2 µg/day (2% of the adult RNI) to as high as 293 µg/day (196% of the adult RNI; see Supplementary Table 4). The EAT-Lancet diet based on Scenario 2 calculations would provide enough iodine to meet the recommended intake for adults in Australia and the Netherlands (141% and 135% of iodine RNI, respectively; Table 3). The primary sources of iodine in the Scenario 2 calculations were dairy products (19.8–80.4 µg/day), fish (1.9–20.5 µg/day) and eggs (4.1–10.1 µg/day; Table 2). In Scenario 2, wholegrains were the main contributor to the iodine content of the EAT-Lancet diet in Australia, the Netherlands and New Zealand (138.2 µg/day, 125.5 µg/day, and 101.6 µg/day, respectively; Table 2) due to iodised salt added to the bread-making process in these countries.

**Fig. 2:**
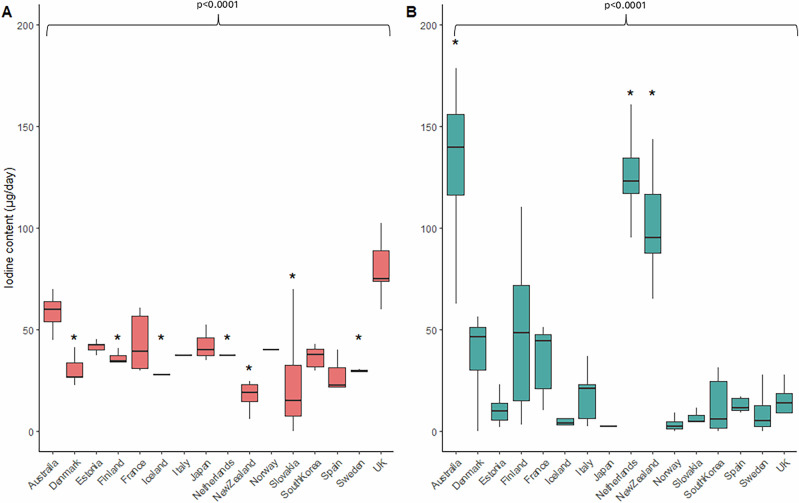
Iodine content per day provided by EAT-Lancet diet. Iodine content per day (µg/day) from **A** dairy foods and **B** wholegrains across the listed countries based on Scenario 2 calculation (i.e. Adapted EAT-Lancet diet to include a wider variety of food codes). *P* value denotes the difference in iodine content between countries (Kruskal–Wallis H test). Statistical significance (*p* < 0.05) compared to the UK is marked with an asterisk (*).

The iodine content of the main contributors to iodine in the EAT-Lancet diet varied between countries (Fig. 2). Iodine content in the dairy products group varied significantly across the countries (*p* < 0.0001) included in this analysis. The iodine content of dairy products was the highest in the UK (80.4 µg/day). Denmark, Finland, Iceland, the Netherlands, New Zealand, Slovakia, and Sweden had significantly lower dairy iodine content when compared to the UK (*p* < 0.0001). Iodine concentrations in wholegrains also varied widely (*p* < 0.0001), with relatively low concentration in the UK wholegrains but significantly higher wholegrain iodine concentration in Australia, New Zealand, and the Netherlands.

### Scenario 3: iodine provision from the vegan EAT-Lancet diet

Once animal-source foods were removed from the EAT-Lancet diet (Scenario 3; Fig. 1), Australia and the Netherlands had the highest total iodine intake following a vegan version of the EAT-Lancet diet with 139.7 and 139.5 µg/day, both providing 93% of the adult RNI (Table 3). By contrast, Japan, Iceland, and Spain had the lowest total iodine intake following a vegan version of the EAT-Lancet diet, contributing only 6%, 7%, and 10% of the adult RNI, respectively (Table 3). Without animal-source food, whole grains provided the largest contribution to the iodine content of the EAT-Lancet diet in countries that used iodised salt in bread – Australia (99%), Denmark (94%), the Netherlands (90%), and New Zealand (95%), providing 138.2, 37.4, 125.5 and 101.6 µg/day, respectively. Vegetables and fruits contributed significantly less to the total iodine intake than whole grains. For instance, Finland had a relatively high iodine contribution from vegetables (11.7 µg/day) and Estonia from fruits (3.7 µg/day), but these still only cover a small proportion of the RNI.

## Discussion

These results highlight the critical need to consider micronutrient adequacy, particularly iodine, when designing global dietary recommendations such as the EAT-Lancet diet^1^. Our findings show that the impact of adopting this diet on iodine intake varies significantly between countries, primarily due to differences in the presence of iodine-fortified plant-based foods, such as bread, and the iodine content of dairy products, which is heavily influenced by farming practices. The EAT-Lancet diet will likely provide inadequate iodine in most countries, underscoring the necessity of adapting dietary guidelines to local nutritional contexts and ensuring contingency plans, such as iodine fortification, are in place.

Across all countries examined, dairy products were the primary source of iodine in the EAT-Lancet diet, providing between 10 and 76 μg/day (7–51% of the adult RNI) from 250 g of dairy products. However, the iodine content of dairy products varies by season^11^, farming practices (e.g. organic vs. conventional)^23^, and country^11^. The upper intake range of dairy foods may meet or exceed iodine requirements in nations with high dairy-iodine concentrations, such as the UK and France. Conversely, in countries with low iodine levels in dairy products, such as New Zealand, South Korea, or Slovakia, even high dairy consumption may not ensure adequate iodine intake. Notably, milk consumption in many European countries has been declining for several decades, even prior to the publication of the EAT-Lancet diet, due to changing dietary preferences and the increasing popularity of plant-based alternatives. This suggests that population-level iodine intake from dairy products may already be decreasing independently of dietary shifts toward sustainability, potentially compounding the risk of iodine deficiency when adopting the EAT-Lancet diet. Additionally, given the EAT-Lancet Commission’s emphasis on reducing animal-source foods, countries with traditionally low dairy consumption are unlikely to increase intake, further elevating the risk of iodine deficiency.

The contribution of fish to iodine intake in the EAT-Lancet diet also varies depending on the type and quantity consumed. While the Commission prioritises oily fish for its omega-3 fatty acid content, the iodine content of white fish is generally higher and may be a better option for meeting iodine needs^24^. However, the recommended fish intake (196 g/week) is higher than current consumption levels in many countries. In 2018, 47% of the global population did not consume at least two servings of seafood per week^25^, making adherence to this recommendation challenging, particularly in countries like the UK, where fish consumption is declining^26^. Eggs provide another source of iodine within the EAT-Lancet diet, contributing ~25 μg per egg^27^. However, the recommended egg intake (13 g/day) provides only 1–7% of the adult RNI. Global egg consumption in 2018 averaged 21 g/day^25^, almost double the EAT-Lancet diet’s recommendation, so adhering to the diet would require a reduction in egg intake, further limiting iodine intake from this source.

Although not included in the EAT-Lancet framework, certain plant-based foods may provide alternative sources of iodine. Seaweed, for example, is naturally high in iodine^28^ and plays a significant role in the diets of some East Asian countries, such as Japan and South Korea, where it is a culturally accepted and frequently-consumed food. In these regions, seaweed can substantially contribute to iodine intake and help achieve iodine sufficiency through sustainable, locally available sources. However, seaweed also has several limitations as an iodine source. Its iodine content varies significantly between types; kelp, in particular, can contain very high levels that may exceed the upper limit of iodine^28^, and its bioavailability may be reduced due to the food matrix^29^. Overconsumption of some types of seaweed has been linked to iodine excess and thyroid dysfunction^30^. Therefore, while seaweed may help address iodine gaps in certain populations, particularly where it is traditionally consumed, it is not a universally dependable or practical solution for ensuring iodine sufficiency at the population level.

Plant-based milk alternatives (e.g. soya, oat, almond milk) are excluded from the EAT-Lancet diet, which focuses on minimally processed foods^1^. However, in countries where these alternatives are fortified with iodine, they may serve as an important source of iodine for those who avoid dairy products^31^. The availability and regulation of iodine fortification in plant-based milks vary widely across countries^32–34^. Where fortification is not mandatory or consistently applied, relying on such products may not guarantee adequate iodine intake, especially if intake is low^12,35^.

While iodine was not explicitly modelled in the EAT-Lancet diet, the Commission has acknowledged concerns regarding potential deficiencies in other micronutrients, such as riboflavin, calcium, and vitamin B12 (particularly in vegetarian/vegan versions of the EAT-Lancet diet)^1^. Our findings, along with those of others^36^, reinforce the concern of micronutrient deficiency associated with the EAT-Lancet diet, and highlight the need for iodine-fortified foods or supplements, particularly in countries where dairy products have a low iodine concentration. In some countries, existing fortification policies may mitigate iodine inadequacy in sustainable diets. For example, in countries with mandatory iodised salt policies, such as Italy, adult iodine requirements could be met solely through the use of iodised salt. According to WHO guidelines (5 g of salt per day fortified with 20–40 mg iodine/kg), iodised salt can provide 100–200 µg of iodine daily, sufficient to meet the recommended intake levels. Conversely, in countries where salt iodisation is voluntary or absent, populations are more reliant on food sources to obtain sufficient iodine^10^. In these settings, the EAT-Lancet diet would be unlikely to meet iodine requirements without additional fortification strategies. Similarly, in Australia and the Netherlands, where iodised salt is used in bread making^10^, the EAT-Lancet diet could provide sufficient iodine to meet the RNI if bread is consumed. In these regions, bread is an important plant-based source of iodine^37,38^. However, in countries like New Zealand, due to the low iodine concentration of cow’s milk^11^, even iodised salt in bread products would be insufficient to meet iodine requirements, necessitating additional strategies, such as fortified foods or increased iodine concentration in dairy products, to ensure adequate iodine intake when following the EAT-Lancet diet.

From an environmental perspective, the EAT-Lancet Commission cautions that widespread adoption of the EAT-Lancet diet could still exceed planetary boundaries unless food production practices and waste reduction are optimised^1^. Consequently, the Commission recommends minimising animal-source food intake, with dairy product consumption suggested at 250 g/day but with a possible range from 0 to 500 g/day. The lower end of this range is encouraged to align with planetary health goals. A vegan diet, composed solely of plant-based products, is thus considered sustainable and recommendable within the EAT-Lancet framework. Without sufficient plant-based sources of iodine and fortification measures, a widespread shift to a vegan version of the EAT-Lancet diet could lead to an increased risk of iodine deficiency, with the iodine provision in some countries potentially falling below a quarter of the adult RNI. A recent study questioned the impact of the diet’s zero-consumption recommendations on micronutrient adequacy, particularly for women of reproductive age in low- and middle-income countries^39^. The study found that adherence to the EAT-Lancet diet was negatively associated with overall micronutrient adequacy without minimum intake values for specific food categories^39^. These findings suggest that caution is warranted before promoting the EAT-Lancet diet as a global health solution.

Several methodological considerations must be acknowledged regarding the calculation of the iodine content in the EAT-Lancet diet. First, cross-country comparisons are complicated by variations in dietary data collection methods, with only harmonised methodologies allowing for direct comparisons. Second, within individual countries, iodine concentrations in foods fluctuate significantly. For example, in the UK, the iodine content of milk varies seasonally, ranging from 50 μg per 250 g in summer to 103 μg in winter^27^, leading to substantial differences in overall iodine content. Third, our iodine content calculations for dairy products are based on liquid milk, potentially underestimating contributions from other dairy products like cheese, which can contain higher iodine concentrations^27^. However, as milk constitutes the majority of dairy product intake^40^, the overall impact is likely to be minimal.

An additional limitation is the reliance on food composition databases, where in some cases, the data used are over 10 years old, which may affect the accuracy of iodine estimates, especially in the dairy foods category. Recent studies have highlighted a decline in the iodine concentration of cow’s milk in the UK^41^ and in other countries^42^, suggesting that older food composition data might overestimate current iodine intake from dairy products. Additionally, the analysis included only 16 countries due to data availability, which limits the generalisability of the findings and could bias results towards countries with more accessible or better-resourced datasets. The inclusion of only English-language sources introduces another potential bias. An updated version of the EAT-Lancet report is expected to be published in late 2025, but major revisions to the diet’s food groups, and therefore iodine content, are not expected and are unlikely to impact our conclusions.

The iodine content estimates in this study do not account for table salt, meaning that in countries with strong salt fortification policies and public-health messaging around iodised salt, actual intake could be considerably higher than estimated.

A final point to note is that iodine intake may be lower than calculated if dietary recommendations are adjusted for energy needs. The EAT-Lancet diet is based on a 2500 kcal/day diet for a 70-kg male or a 60-kg female with moderate to high physical activity. If scaled down to 2000 kcal/day, recommendations for animal-source products, and consequently iodine intake, would decrease.

While this study focuses on adult dietary requirements, the findings have important implications for other population strata, including children and adolescents, who have distinct iodine needs relative to their body size and developmental stage. Children, in particular, are more vulnerable to iodine deficiency due to their high reliance on dairy foods^43,44^ for iodine requirements and the critical role of iodine in growth and cognitive development^45^. If sustainable dietary transitions are adopted at the household level, there is a risk that younger populations could also be exposed to inadequate iodine intake, especially in countries lacking robust iodine fortification strategies.

Although dietary guidelines like the EAT-Lancet diet offer a useful framework for promoting sustainable and health-conscious eating, this study highlights a crucial nutritional gap: the diet may not provide sufficient iodine for adults or pregnant women. Given the Eat-Lancet’s overarching recommendation to reduce animal-source foods, it is essential for consumers and health professionals to recognise the risks associated with plant-based diets, such as for iodine deficiency, which should be balanced against the potential environmental and health benefits. Clear accompanying guidance on fortified foods or supplementation is required to address this.

As this study demonstrates, individuals transitioning to plant-based diets in countries where iodised salt is not widely used in staple products like bread, whether for sustainability or other reasons, may face a heightened risk of iodine deficiency. Therefore, public health strategies must address this potential risk by promoting fortified foods, such as iodised salt, or supplements to ensure that plant-based diets provide adequate iodine intake. Without such measures, the shift towards sustainable eating habits could inadvertently lead to an increased prevalence of iodine insufficiency, particularly among vulnerable groups such as pregnant women, for whom iodine is critical for fetal development and overall health.

## Methods

### Country selection

Countries were included in this analysis if they had a national food composition table (with an English language translation) that contained iodine concentration data for all EAT-Lancet diet food groups. Online food composition tables were consulted to collate the most up-to-date information on the iodine concentration from countries worldwide. Firstly, the European Food Information Resource website was used to compile a list of food composition tables^19^. This was cross-referenced with two published reviews that indicated which tables included the iodine content of foods^18,46^ to create a final list of countries with online food composition tables that included iodine values (Table 1).

### Values from the EAT-Lancet diet

The EAT-Lancet diet provides daily food intake recommendations for a diet designed to optimise human health and environmental sustainability as described in the EAT-Lancet report^1^. The EAT-Lancet diet was designed to meet the WHO global intake recommendations for all nutrients, except phosphorus and copper, where the National Academy of Medicine (NAM, formerly IoM) recommendations were used^21^. The EAT-Lancet diet takes a global focus and includes broadly global foods from eight food groups: (i) fruit, (ii) vegetables, (iii) starchy vegetables/tubers, (iv) whole grains, (v) dairy foods, (vi) protein sources (including meat and alternatives), (vii) added fats and (viii) added sugar. The recommendations provide a target based on an average amount (shown in Table 2), as well as lower and upper boundaries for each food group listed (e.g. 250 g dairy foods/day with a range of 0–500 g, shown in Supplementary Table 1). The EAT-Lancet diet was created and modelled using a single list of 35 commonly-consumed foods in the United States^47^, and the nutrient composition of the diet was estimated by the EAT-Lancet Commission using the U.S. Department of Agriculture (USDA) Foods Database, FoodData Central^48^ (Supplementary Table 1). However, the EAT-Lancet Commission did not use food composition data from other countries and did not consider iodine, as the USDA database does not include iodine values.

### Food composition tables: search strategy and data extraction

The selected food composition tables were searched using the food-item terms used by the EAT-Lancet commission^1^, for example, “whole milk”, “Atlantic cod”, and “raw brown rice” (Supplementary Table 1). Food composition tables were searched in July 2023. In cases where an exact match for an EAT-Lancet food group was not available, the closest equivalent item available in the national food composition table was selected based on similarity in food type and form. The data extracted included exact food names, database food codes and iodine content (μg/100 g).

### Scenarios for calculating iodine provided by EAT-Lancet diet

Our study used three scenarios to calculate the amount of iodine provided by the EAT-Lancet diet, as well as a vegan version of the diet. Iodised table salt was excluded from the quantitative analysis due to a lack of harmonised and comprehensive data on iodine content and use across countries; iodised salt policies in the sixteen countries in our study are listed in Supplementary Table 2.

### Scenario 1: Iodine provision from the original EAT-Lancet diet

As a first step, individual food items that were the closest match to 35 foods used in the EAT-Lancet diet (Supplementary Table 1)^47^ were selected from the country-specific food composition databases; the iodine concentration was extracted and adjusted to the food weight recommended in the EAT-Lancet diet. For example, the diet recommends 28 g/day of fish, based on 14 g/day of sockeye salmon and 14 g/day of Atlantic cod; therefore, these fish types and proportional weights were also used in our study for the Scenario 1 calculations. A total daily iodine intake was summed from the individual food items, proportional to the EAT-Lancet diet.

### Scenario 2: iodine provision from an adapted EAT-Lancet diet

Scenario 2 maintained the overall proportions of each food group and upheld the principles of the EAT-Lancet diet (i.e. an emphasis on primarily whole and minimally processed foods). However, this scenario was designed to better reflect the diversity and variability of real-world dietary patterns by incorporating a wider range of foods within each EAT-Lancet food group. All relevant food items listed under each group in the national food composition tables were included and equally weighted, provided they met the inclusion criteria. This included both raw and cooked forms of foods, where appropriate, and a range of preparation methods such as boiling, steaming, baking, grilling, and microwaving.

For example, in Scenario 1, the ‘whole grains’ group was limited to “wheat, hard red spring” and “brown rice, long grain, raw”. In contrast, Scenario 2 expanded this group to include all available whole-grain items from the national food tables, such as different types of rice (e.g. white, brown), flours, breads, and cereals. Similarly, other food groups (e.g. vegetables, legumes, or dairy) included multiple food types and forms where iodine data were available. Fortified bread products were also included in this scenario, where they appeared in the national food composition tables.

To preserve consistency with the EAT-Lancet diet’s emphasis on whole foods, items with significant added ingredients or those prepared with iodine-containing components (e.g. foods cooked in milk, cream-based sauces, or commercial ready meals) were excluded to avoid confounding from cross-contamination. Although fortified plant-based milk alternatives (e.g. soya or oat milk) are increasingly common^32^, these products were not included in this scenario due to the inconsistent availability of iodine data for these products across national databases.

This scenario was intended to simulate the impact of broader food choice within dietary patterns and to assess how such variability might influence iodine intake, even when adhering to the EAT-Lancet food group framework.

### Scenario 3: iodine provision from the vegan EAT-Lancet diet

The EAT-Lancet Commission encourages animal-source food intake to be as close to zero as possible to ensure greenhouse gas emissions are within planetary boundaries. Therefore, in this scenario, the results for Scenario 2 calculations are presented without animal-source foods to represent a vegan version of the EAT-Lancet diet.

### Statistical analysis

The total daily iodine intake provision from the dietary recommendations was calculated and compared to iodine intake recommendations for adults and during pregnancy. We used several intake recommendations for comparison, primarily using the values equivalent to the RNI as we calculated iodine provision of the diet (i.e. at an individual level), not the population average intake, for which the Estimated Average Requirement would be more appropriate^49^. For adults, we compared the iodine provision of the diet against the recommended intake of 150 µg/day, as defined by three major authorities: the RNI from the WHO^20^, the Recommended Dietary Allowance from the United States NAM^50^, and the Adequate Intake (AI) set by the European Food Safety Authority^22^. For pregnancy, we present the comparison as a range to account for variation in iodine recommendations around the world, from 200 µg/day in Europe^22^, 220 µg/day in the USA^50^, and 250 µg/day according to WHO^20^. The normality of data was assessed by Shapiro–Wilk test, and differences in iodine intake between countries were evaluated using the Kruskal–Wallis H test, with Bonferroni correction for multiple testing. All analyses were carried out in R studio version 2023.03.1+446^51^, *p* < 0.05 was set to denote statistical significance.

## Supplementary information

Supplementary Information

## Data Availability

The datasets analysed during the current study are publicly available and can be found in the manuscript (Table 1). The underlying code for this study is not publicly available but may be made available to qualified researchers on reasonable request from the corresponding author.

## Code availability

The underlying code for this study is not publicly available but may be made available to qualified researchers on reasonable request from the corresponding author.
