# Supplementary material for: Eating within planetary boundaries - a cross-country analysis of iodine provision from the EAT-Lancet diet
Source: NPJ Sci Food. 2025 Nov 24;9:252. doi: 10.1038/s41538-025-00612-7 (PMC12644469; doi:10.1038/s41538-025-00612-7)
Supplement: Supplementary file 1 — Supplementary Information [file 41538_2025_612_MOESM1_ESM.docx]

Supplementary Table 1. USDA food codes and quantities used by the EAT-Lancet commission when modelling the EAT-Lancet diet and possible ranges for an intake of 2500kcal/day^47^.

| Foods | USDA code | Food intake (g/day) | Possible intake range (g/day) |
| --- | --- | --- | --- |
| Whole grains | | |  |
| Wheat, hard red spring | 20071.0 | 116.0 | - |
| Rice, brown, long-grain, raw | 20036.0 | 116.0 |  |
| Tubers or starchy vegetables | | |  |
| Potato, flesh & skn, raw | 11352.0 | 50.0 | 0.0-100.0 |
| Vegetables | | |  |
| Raw spinach | 11457.0 | 100.0 | 200.0-600.0 |
| Baked, unsalted winter squash | 11644.0 | 33.0 |  |
| Boiled, drained, unsalted carrots | 11125.0 | 33.0 |  |
| Ripe, red tomatoes | 11529.0 | 34.0 |  |
| Raw, onions | 11282.0 | 33.0 |  |
| Boiled, drained, unsalted summer squash | 11478.0 | 33.0 |  |
| Raw green peppers | 11333.0 | 34.0 |  |
| Fruit | | |  |
| Raw apples with skin | 9003.0 | 66.0 | 100.0-300.0 |
| Raw oranges | 9200.0 | 66.0 |  |
| Raw bananas | 9040.0 | 66.0 |  |
| Dairy foods | | |  |
| Whole milk | 1077.0 | 250.0 | 0-500.0 |
| Protein sources | | |  |
| Beef, ground, 85% ln meat / 15% fat, raw | 23567.0 | 7.0 | 0.0-14.0 |
| Chicken, broilers or fryers, meat & skn, raw | 5006.0 | 29.0 | 0.0-58.0 |
| Pork, frsh, comp (leg, loin, shldr, & spareribs) | 10187.0 | 7.0 | 0.0-14.0 |
| Raw whole egg | 1123.0 | 13.0 | 0.0-25.0 |
| Dry heat cooked sockeye salmon | NA | 14.0 | 0.0-100.0 |
| Dry heat cooked atlantic cod | NA | 14.0 |  |
| Legumes | | |  |
| Lentils, raw | 16069.0 | 25.0 | 0.0-100.0 |
| Beans, navy, mature seeds, raw | 16037.0 | 25.0 |  |
| Peanuts, all types, raw | 16087.0 | 25.0 | 0.0-75.0 |
| Oil roasted, unsalted almonds | 12065.0 | 12.5 | - |
| Oil roasted, unsalted cashews | 12086.0 | 12.5 |  |
| Soybeans, mature seeds, raw | 16108.0 | 25.0 | 0.0-50.0 |
| Added fats | | |  |
| Oil, vegetable, palm | 4055.0 | 6.8 | 0.0-6.8 |
| Oil, soybean, salad or cooking | 4044.0 | 8.0 | 20.0-80.0 |
| Vegetable oil, canola | 4582.0 | 8.0 |  |
| Oil, olive, salad or cooking | 4053.0 | 8.0 |  |
| Oil, vegetable, sunflower, linoleic, | 4506.0 | 8.0 |  |
| Oil, peanut, salad or cooking | 4042.0 | 8.0 |  |
| Salted butter | 1001.0 | 0.0 | - |
| Lard | 4002.0 | 4.0 | 0.0-5.0 |
| Added sugar | | |  |
| Granulated sugar | 19335.0 | 31.0 | 0.0-31.0 |

NA: data not available

*Supplementary Table 2. Countries with mandatory, voluntary and no/unknown legislation or regulation for salt iodization.*

| Country | Iodised salt policy | Notes |
| --- | --- | --- |
| Australia | Voluntary^66^ | Mandatory for the production of bread and bakery products |
| Denmark | Mandatory^10^ | Mandatory for household salt |
| Estonia | Unknown^10^ | NA |
| Finland | Voluntary^10^ | NA |
| France | Voluntary^10^ | NA |
| Iceland | No policy^10^ | NA |
| Italy | Mandatory^10^ | Shops must sell both iodised and non-iodised salt, but are required to only provide non-iodised salt on specific request |
| Japan | No policy^67^ | NA |
| Netherlands | Voluntary^10^ | Mandatory for the production of bread and bakery products |
| New Zealand | Voluntary^66^ | Mandatory for the production of bread and bakery products |
| Norway | Voluntary^10^ | NA |
| Slovakia | Mandatory^10^ | NA |
| Spain | Voluntary^10^ | NA |
| South Korea | No policy^68^ | NA |
| Sweden | Voluntary^10^ | NA |
| UK | No policy^10^ | NA |

|  | Whole grains | Tubers or starchy vegetables | Vegetables | Fruits | Dairy foods | Beef & lamb | Pork | Chicken & other poultry | Eggs | Fish | Legumes | Added fats | Added sugar |
| --- | --- | --- | --- | --- | --- | --- | --- | --- | --- | --- | --- | --- | --- |
| EAT Lancet diet (g/d) | 232  (NA) | 50  (0-100) | 300  (200-600) | 200  (100-300) | 250  (0-500) | 7  (0-14) | 7  (0-14) | 29  (0-58) | 13  (0-25) | 28  (0-100) | 125  (25-225) | 51.8  (20-91.8) | 31  (0-31) |
| Iodine provision from food groups and total (µg/d) | | | | | | | | | | | | | |
| Australia | 27.1  (NA) | 0.0  (0.0-0.0) | 3.6  (2.4-7.3) | 0.3  (0.1-0.4) | 59.0  (0.0-118) | 0.0  (0.0-0.0) | 0.1  (0.0-0.1) | 0.0  (0.0-0.0) | 7.5  (0.0-14.4) | 7.0  (0.0-25.0) | 1.0  (0.3-1.6) | 0.1  (0.1-0.1) | 3.6  (0.0-3.6) |
| Denmark | 6.9  (NA) | 0.6  (0.0-1.2) | 2.0  (1.3-4.0) | 0.2  (0.1-0.3) | 26.8  (0.0-53.5) | 0.0  (0.0-0.0) | 0.1  (0.0-0.1) | 0.3  (0.0-0.5) | 8.5  (0.0-16.4) | 39.4  (0.0-141.0) | 2.6  (0.0-5.2) | 0.2  (0.0-0.2) | 0.9  (0.0-0.9) |
| Estonia | 5.2  (NA) | 0.5  (0.0-1.0) | 2.3  (1.5-4.5) | 3.3  (1.7-5.0) | 47.5  (0.0-95.0) | 0.2  (0.0-0.4) | 0.1  (0.1-0.2) | 1.7  (0.0-3.5) | 5.6  (0.0-10.8) | 30.7  (0.0-109.5) | 4.8  (1.6-9.2) | 0.2  (0.0-0.2) | 0.7  (0.0-0.7) |
| Finland | 9.1  (NA) | 0.5  (0.0-1.0) | 3.0  (2.0-6.0) | 1.4  (0.7-2.1) | 35.5  (0.0-71.0) | 0.2  (0.0-0.4) | 0.2  (0.0-02) | 4.4  (0.0-8.7) | 4.8  (0.0-9.3) | 22.0  (0.0-78.7) | 4.9  (1.9-9.2) | 0.2  (0.0-0.2) | 1.2  (0.0-1.2) |
| France | 3.1  (NA) | 0.6  (0.0-1.2) | 3.8  (2.5-7.5) | 0.0  (0.0-0.0) | 60.8  (0.0-121.5) | 0.1  (0.0-0.1) | 0.1  (0.0-0.1) | 0.1  (0.0-0.2) | 2.7  (0.0-5.3) | 16.6  (0.0-59.1) | 0.5  (0.0-1.0) | 0.2  (0.0-0.2) | 0.4  (0.0-0.4) |
| Iceland | 6.7  (NA) | 0.6  (0.0-1.2) | 3.7  (2.5-7.4) | 0.6  (0.3-0.9) | 28.0  (0.0-56.0) | 0.1  (0.0-0.2) | 0.2  (0.0-0.4) | 0.7  (0.0-1.5) | 4.1  (0.0-7.9) | 10.5  (0.0-37.5) | 0.6  (0.0-1.3) | 2.3  (1.0-4.3) | 0.9  (0.0-0.9) |
| Italy | 0.0  (NA) | 0.5  (0.0-1.0) | 3.0  (2.0-6.0) | 6.7  (3.3-10.0) | 37.5  (0.0-75.0) | 0.7  (0.0-1.4) | 0.4  (0.0-0.7) | 1.7  (0.0-3.5) | 6.9  (0.0-13.3) | 20.6  (0.0-73.5) | 7.4  (1.6-16.5) | 0.3  (0.0-0.3) | 0.0  (0.0-0.0) |
| Japan | 0.0  (NA) | 0.0  (0.0-0.0) | 3.4  (2.3-6.9) | 0.0  (0.0-0.0) | 40.0  (0.0-80.0) | 0.1  (0.0-0.1) | 0.0  (0.0-0.0) | 0.0  (0.0-0.0) | 2.2  (0.0-4.3) | 49.0  (0.0-175.0) | 0.3  (0.0-0.8) | 0.0  (0.0-0.0) | 0.0  (0.0-0.0) |
| Netherlands | 5.6  (NA) | 1.3  (0.0-2.5) | 5.7  (3.8-11.5) | 5.0  (2.5-7.5) | 37.3  (0.0-74.5) | 0.2  (0.0-0.5) | 0.1  (0.0-0.1) | 1.7  (0.0-3.5) | 4.1  (0.0-7.8) | 37.4  (0.0-133.4) | 1.5  (0.6-3.1) | 0.1  (0.0-0.1) | 0.7  (0.0-0.7) |
| New Zealand | 2.6  (NA) | 1.9  (0.0-3.7) | 3.7  (2.5-7.4) | 0.1  (0.1-0.2) | 19.3  (0.0-38.5) | 0.1  (0.0-0.1) | 0.1  (0.0-0.2) | 0.2  (0.0-0.4) | 7.2  (0.0-13.8) | 6.2  (0.0-22.0) | 0.5  (0.2-0.9) | 0.1  (0.0-0.1) | 0.3  (0.0-0.3) |
| Norway | 4.6  (NA) | 0.5  (0.0-1.0) | 5.1  (3.4-10.3) | 4.0  (2.0-6.0) | 40.0  (0.0-80.0) | 0.1  (0.0-0.3) | 0.0  (0.0-0.0) | 0.3  (0.0-0.6) | 4.4  (0.0-8.5) | 39.6  (0.0-141.5) | 5.8  (0.3-11.8) | 0.0  (0.0-0.0) | 0.6  (0.0-0.6) |
| Slovakia | 10.0  (NA) | 4.5  (0.0-9.0) | 11.8  (7.9-23.7) | 10.3  (5.1-15.4) | 10.0  (0.0-20.0) | 0.6  (0.0-1.2) | 0.0  (0.0-0.0) | 0.3  (0.0-0.6) | 12.6  (0.0-24.3) | 21.6  (0.0-77.0) | 10.9  (1.6-24.1) | 0.2  (0.0-0.2) | 1.3  (0.0-1.3) |
| South Korea | 0.0  (NA) | 0.0  (0.0-0.0) | 0.5  (0.4-1.0) | 0.0  (0.0-0.0) | 15.2  (0.0-30.4) | 0.1  (0.0-0.1) | 0.0  (0.0-0.1) | 0.0  (0.0-0.0) | 3.9  (0.0-7.5) | 78.9  (0.0-281.9) | 1.6  (0.1-3.5) | 0.0  (0.0-0.0) | 0.0  (0.0-0.0) |
| Spain | 1.3  (NA) | 1.3  (0.0-2.6) | 12.5  (8.3-25.0) | 2.7  (1.3-4.0) | 21.5  (0.0-43.0) | 0.1  (0.0-0.1) | 0.2  (0.0-0.4) | 0.5  (0.0-1.0) | 2.6  (0.0-5.0) | 27.8  (0.0-99.2) | 6.2  (0.3-16.8) | 0.2  (0.0-0.2) | 0.2  (0.0-0.2) |
| Sweden | 3.0  (NA) | 0.7  (0.0-1.3) | 3.6  (2.4-7.1) | 2.7  (1.3-4.0) | 29.5  (0.0-59.0) | 2.6  (0.0-5.1) | 0.1  (0.0-0.2) | 0.1  (0.0-0.2) | 5.5  (0.0-10.5) | 15.7  (0.0-56.0) | 10.0  (0.0-20.0) | 0.1  (0.0-0.1) | 0.4  (0.0-0.4) |
| UK | 0.0  (NA) | 0.0  (0.0-0.0) | 6.4  (4.3-12.9) | 4.7  (2.3-7.0) | 76.3  (0.0-152.5) | 0.6  (0.0-1.2) | 0.0  (0.0-0.0) | 1.5  (0.0-2.9) | 6.5  (0.0-12.5) | 24.5  (0.0-87.5) | 6.6  (1.6-13.5) | 0.6  (0.0-0.6) | 1.4  (0.0-1.4) |
| Average | **5.3**  (NA) | **0.8**  **(0.0-1.7)** | **6.4**  **(3.1-9.3)** | **2.6**  **(1.3-3.9)** | **36.5**  **(0.0-73.0)** | **0.4**  **(0.0-0.7)** | **0.1**  **(0.0-0.2)** | **0.8**  **(0.0-1.7)** | **5.6**  **(0.0-10.7)** | **28.0**  **(0.0-99.9)** | **3.7**  **(0.6-8.7)** | **0.3**  **(0.1-0.4)** | **0.6**  **(0.0-0.6)** |

Supplementary Table 3. Scenario 1 calculations of the mean and ranges iodine content of each food group in the EAT-Lancet diet (µg/d): Food codes matched to EAT-Lancet diet

*Iodine intake recommendations for adults: 150µg/day^20-22^

**Iodine intake recommendations for pregnancy: 200-250µg/d

Supplementary Table 4. Scenario 2 calculations of the mean and ranges of iodine content of each food group in the EAT-Lancet diet (µg/day): EAT-Lancet diet adapted to include a wider variety of food codes.

|  | Whole grains | Tubers or starchy vegetables | Vegetables | Fruits | Dairy foods | Beef & lamb | Pork | Chicken & other poultry | Eggs | Fish | Legumes | Added fats | Added sugar |
| --- | --- | --- | --- | --- | --- | --- | --- | --- | --- | --- | --- | --- | --- |
| EAT Lancet diet (g/d) | 232  (NA) | 50  (0-100) | 300  (200-600) | 200  (100-300) | 250  (0-500) | 7  (0-14) | 7  (0-14) | 29  (0-58) | 13  (0-25) | 28  (0-100) | 125  (25-225) | 51.8  (20-91.8) | 31  (0-31) |
| Iodine provision from food groups and total (µg/d) | | | | | | | | | | | | | |
| Australia | 138.2 | 0.1  (0.0-0.1) | 1.2  (0.8-2.3) | 0.1  (0.0-0.1) | 59.3  (0.0-118.7) | 0.1  (0.0-0.2) | 0.1  (0.0-0.1) | 0.5  (0.0-0.9) | 10.0  (0.0-19.2) | 1.9  (0.0-13.5) | 0.1  (0.0-0.1) | 0.0  (0.0-0.0) | 0.0  (0.0-0.0) |
| Denmark | 37.4 | 0.2  (0.0-0.3) | 1.6  (1.1-3.2) | 0.2  (0.1-0.2) | 29.8  (0.0-59.6) | 0.1  (0.0-0.2) | 0.1  (0.0-0.3) | 1.2  (0.0-2.3) | 4.4  (0.0-8.4) | 12.4  (0.0-88.6) | 0.2  (0.0-0.4) | 0.0  (0.0-0.0) | 0.0  (0.0-0.0) |
| Estonia | 19.7 | 0.6  (0.0-1.1) | 5.1  (3.4-10.3) | 3.7  (1.8-5.5) | 42.2  (0.0-84.3) | 0.2  (0.0-0.4) | 0.2  (0.0-0.3) | 0.9  (0.0-1.8) | 5.7  (0.0-11.0) | 10.4  (0.0-73.9) | 0.6  (0.2-1.0) | 0.0  (0.0-0.0) | 0.0  (0.0-0.0) |
| Finland | 44.5 | 0.5  (0.0-1.0) | 11.7  (7.8-23.4) | 1.8  (0.9-2.7) | 35.7  (0.0-71.5) | 1.0  (0.0-2.0) | 1.0  (0.0-2.0) | 6.6  (0.0-13.3) | 4.6  (0.0-8.9) | 10.0  (0.0-71.6) | 0.9  (0.6-1.4) | 0.0  (0.0-0.0) | 1.6  (0.0-1.6) |
| France | 35.5 | 1.4  (0.0-2.8) | 5.2  (2.5-7.5) | 2.2  (1.1-3.3) | 43.3  (0.0-86.7) | 0.3  (0.0-0.6) | 0.2  (0.0-0.3) | 3.6  (0.0-7.3) | 6.3  (0.0-12.1) | 8.4  (0.0-59.8) | 0.7  (0.2-1.3) | 0.0  (0.0-0.0) | 0.1  (0.0-0.1) |
| Iceland | 5.2 | 0.6  (0.0-1.2) | 2.8  (1.9-5.7) | 0.6  (0.3-0.9) | 28.0  (0.0-56.0) | 0.1  (0.0-0.2) | 0.2  (0.0-0.4) | 0.7  (0.0-1.3) | 4.1  (0.0-7.9) | 4.9  (0.0-35.2) | 0.4  (0.0-0.8) | 0.4  (0.2-0.7) | 0.0  (0.0-0.0) |
| Italy | 22.9 | 0.5  (0.0-1.0) | 3.0  (2.0-6.0) | 10.0  (5.0-15.0) | 37.5  (0.0-75.0) | 0.8  (0.0-1.5) | 0.4  (0.0-0.8) | 2.0  (0.0-4.1) | 6.9  (0.0-13.3) | 9.9  (0.0-70.6) | 2.2  (0.4-5.5) | 0.0  (0.0-0.0) | 0.0  (0.0-0.0) |
| Japan | 2.3 | 0.0  (0.0-0.0) | 3.7  (2.4-7.3) | 0.0  (0.0-0.0) | 42.5  (0.0-85.0) | 0.1  (0.0-0.1) | 0.1  (0.0-0.2) | 0.7  (0.0-1.5) | 2.1  (0.0-4.0) | 13.2  (0.0-94.3) | 0.1  (0.0-0.2) | 0.0  (0.0-0.0) | 2.3  (0.0-2.3) |
| Netherlands | 125.5 | 1.3  (0.0-2.5) | 7.2  (4.8-14.5) | 5.0  (2.5-7.5) | 37.3  (0.0-74.5) | 0.3  (0.0-0.6) | 0.2  (0.0-0.4) | 1.6  (0.0-3.1) | 4.3  (0.0-8.4) | 19.8  (0.0-141.3) | 0.5  (0.2-0.8) | 0.0  (0.0-0.0) | 0.0  (0.0-0.0) |
| New Zealand | 101.6 | 0.4  (0.0-0.8) | 4.0  (2.7-8.0) | 0.3  (0.1-0.4) | 19.8  (0.0-39.7) | 0.1  (0.0-0.2) | 0.1  (0.0-0.2) | 0.7  (0.0-1.4) | 7.2  (0.0-13.9) | 2.4  (0.0-17.0) | 0.3  (0.0-0.6) | 0.0  (0.0-0.0) | 0.0  (0.0-0.0) |
| Norway | 4.0 | 0.8  (0.0-1.5) | 7.4  (4.9-14.8) | 4.5  (2.3-6.8) | 40.1  (0.0-80.2) | 0.2  (0.0-0.3) | 0.0  (0.0-0.1) | 0.5  (0.0-1.0) | 4.7  (0.0-9.0) | 20.5  (0.0-146.7) | 1.2  (0.2-2.3) | 0.0  (0.0-0.0) | 0.0  (0.0-0.0) |
| Slovakia | 7.0 | 3.7  (0.0-7.4) | 15.7  (10.4-31.3) | 10.3  (5.1-15.4) | 25.0  (0.0-50.0) | 0.8  (0.0-1.6) | 0.1  (0.0-0.2) | 1.6  (0.0-3.2) | 9.8  (0.0-18.8) | 10.6  (0.0-75.8) | 2.3  (0.5-5.1) | 0.5  (0.2-0.9) | 0.0  (0.0-0.0) |
| South Korea | 11.4 | 0.0  (0.0-0.0) | 1.3  (0.9-2.7) | 0.0  (0.0-0.0) | 36.3  (0.0-72.7) | 0.0  (0.0-0.1) | 0.1  (0.0-0.3) | 1.7  (0.0-3.4) | 10.1  (0.0-19.4) | 12.3  (0.0-87.7) | 0.7  (0.0-1.5) | 0.0  (0.0-0.0) | 0.0  (0.0-0.0) |
| Spain | 12.4 | 1.1  (0.0-2.1) | 8.2  (5.5-16.4) | 2.7  (1.3-4.0) | 28.0  (0.0-56.0) | 0.3  (0.0-0.7) | 0.1  (0.0-0.3) | 1.5  (0.0-3.0) | 5.7  (0.0-10.9) | 9.8  (0.0-70.3) | 1.3  (0.1-3.2) | 0.0  (0.0-0.0) | 0.0  (0.0-0.0) |
| Sweden | 8.3 | 1.4  (0.0-2.9) | 6.4  (4.2-12.7) | 1.1  (0.6-1.7) | 29.8  (0.0-59.6) | 0.3  (0.0-0.5) | 0.1  (0.0-0.3) | 0.3  (0.0-0.6) | 5.6  (0.0-10.9) | 13.4  (0.0-95.7) | 2.5  (0.0-2.9) | 0.0  (0.0-0.0) | 0.0  (0.0-0.0) |
| UK | 15.0 | 0.6  (0.0-1.1) | 7.7  (5.2-15.5) | 6.0  (3.0-9.0) | 80.4  (0.0-160.7) | 0.8  (0.0-1.6) | 0.5  (0.0-1.1) | 1.7  (0.0-3.4) | 7.2  (0.0-13.9) | 12.5  (0.0-89.3) | 1.8  (0.3-4.0) | 0.0  (0.0-0.0) | 0.0  (0.0-0.0) |
| Average | **36.9** | **0.8**  **(0.0-1.6)** | **5.8**  **(3.9-11.6)** | **3.0**  **(1.5-4.5)** | **38.4**  **(0.0-76.9)** | **0.3**  **(0.0-0.7)** | **0.2**  **(0.0-0.5)** | **1.6**  **(0.0-3.2)** | **6.2**  **(0.0-11.9)** | **10.8**  **(0.0-77.0)** | **1.0**  **(0.2-2.1)** | **0.1**  **(0.0-0.1)** | **0.3**  **(0.0-0.3)** |

*Iodine intake recommendations for adults: 150µg/day^20-22^

**Iodine intake recommendations for pregnancy: 200-250µg/day^20-22^
